# Striking Discordance Between Morphological Residual Disease and Histological Pathological Complete Response: A Case Report of Pembrolizumab Treatment in Mismatch Repair-Deficient Colorectal Cancer With Liver Metastases

**DOI:** 10.7759/cureus.114704

**Published:** 2026-08-18

**Authors:** Kohei Ito, Kengo Shibata, Nobuki Ichikawa, Tadashi Yoshida, Akinobu Taketomi

**Affiliations:** 1 Department of Gastroenterological Surgery I, Hokkaido University Graduate School of Medicine, Sapporo, JPN

**Keywords:** colorectal cancer, dmmr, liver metastasis, pathological complete response, pembrolizumab

## Abstract

Immune checkpoint inhibitors (ICIs) have demonstrated high response rates in mismatch repair-deficient (dMMR) colorectal cancer (CRC). However, radiological or macroscopic findings may suggest residual tumor even when a pathological complete response (pCR) has been achieved. We report a case of dMMR CRC with liver metastases in which pCR was confirmed in both the primary tumor and metastatic lesions after preoperative pembrolizumab therapy. A 70-year-old man with diabetes mellitus and hypertension presented with anemia and was diagnosed with sigmoid colon cancer. Further evaluation revealed cStage IVa dMMR CRC with multiple liver metastases. Laparoscopic transverse colostomy was performed for tumor-related obstruction. Although resection of both the primary tumor and liver metastases was initially considered, treatment of ischemic heart disease with percutaneous coronary intervention was prioritized, and pembrolizumab was administered for six courses during this period. After marked reduction of the primary tumor and liver metastases, robot-assisted sigmoid colectomy followed by laparoscopic right hepatectomy was performed. Although preoperative imaging and intraoperative findings suggested residual tumor, pathological examination revealed no viable cancer cells at either site. This case highlights the discrepancy between morphological residual disease and pathological response after ICI therapy and underscores the importance of comprehensive response assessment.

## Introduction

Colorectal cancer (CRC) remains one of the leading causes of cancer-related death in Japan and is associated with high incidence and mortality. Particularly in advanced and metastatic cases, treatment selection significantly impacts patient prognosis and quality of life (QOL), making personalized therapy based on molecular characteristics highly desirable. Mismatch repair-deficient (dMMR) CRC, a distinct subtype representing approximately 4-5% of metastatic CRC cases, is characterized by impaired DNA mismatch repair, leading to a high tumor mutational burden (TMB) and enhanced immunogenicity [1,2]. These tumors exhibit remarkable sensitivity to immune checkpoint inhibitors (ICIs), which restore antitumor immune activity by blocking inhibitory immune signaling pathways [3-5].

Initially, ICI therapy was primarily indicated for unresectable or recurrent disease; however, neoadjuvant use has shown high pathological response rates in resectable non-metastatic dMMR CRC [5,6]. In contrast, evidence in stage IV disease with resectable distant metastases remains limited. Recent reports have also described pathological complete response (pCR), defined as the absence of viable tumor cells in resected specimens, despite persistent abnormalities on imaging or endoscopic findings [7,8]. Here, we report a case of stage IVa dMMR sigmoid colon cancer with multiple liver metastases in which preoperative pembrolizumab achieved pCR in both the primary tumor and liver metastases despite persistent morphologically suspicious lesions.

## Case presentation

**Figure 1:**
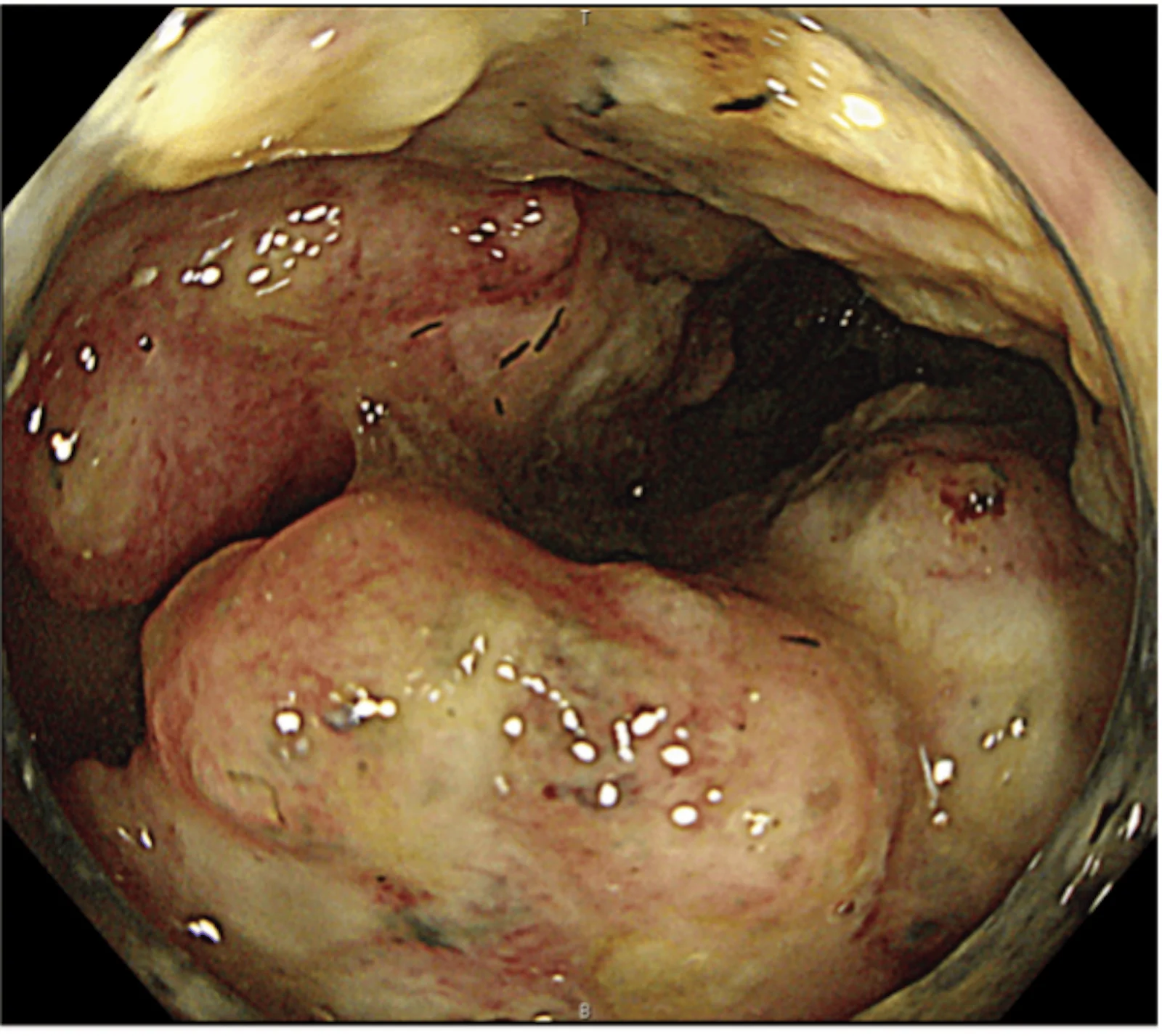
Lower gastrointestinal endoscopic image before pembrolizumab administration A circumferential type 2 tumor was observed in the sigmoid colon, 15 cm from the anal verge.

**Figure 2:**
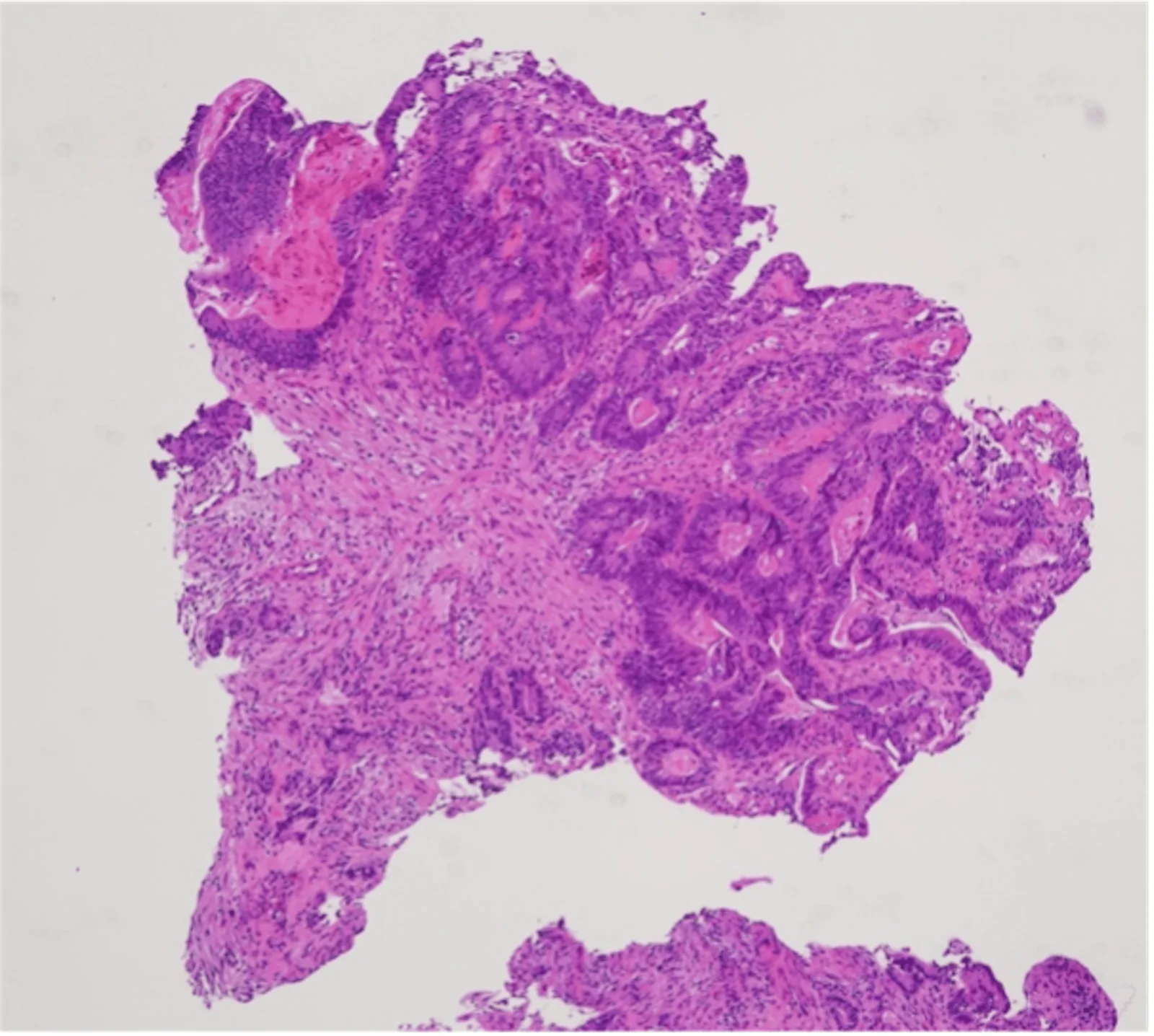
Hematoxylin and eosin (H&E) staining of the primary lesion (H&E, ×40) H&E staining revealed proliferation of neoplastic glands showing loss of polarity, irregular branching, and fusion.

**Figure 3:**
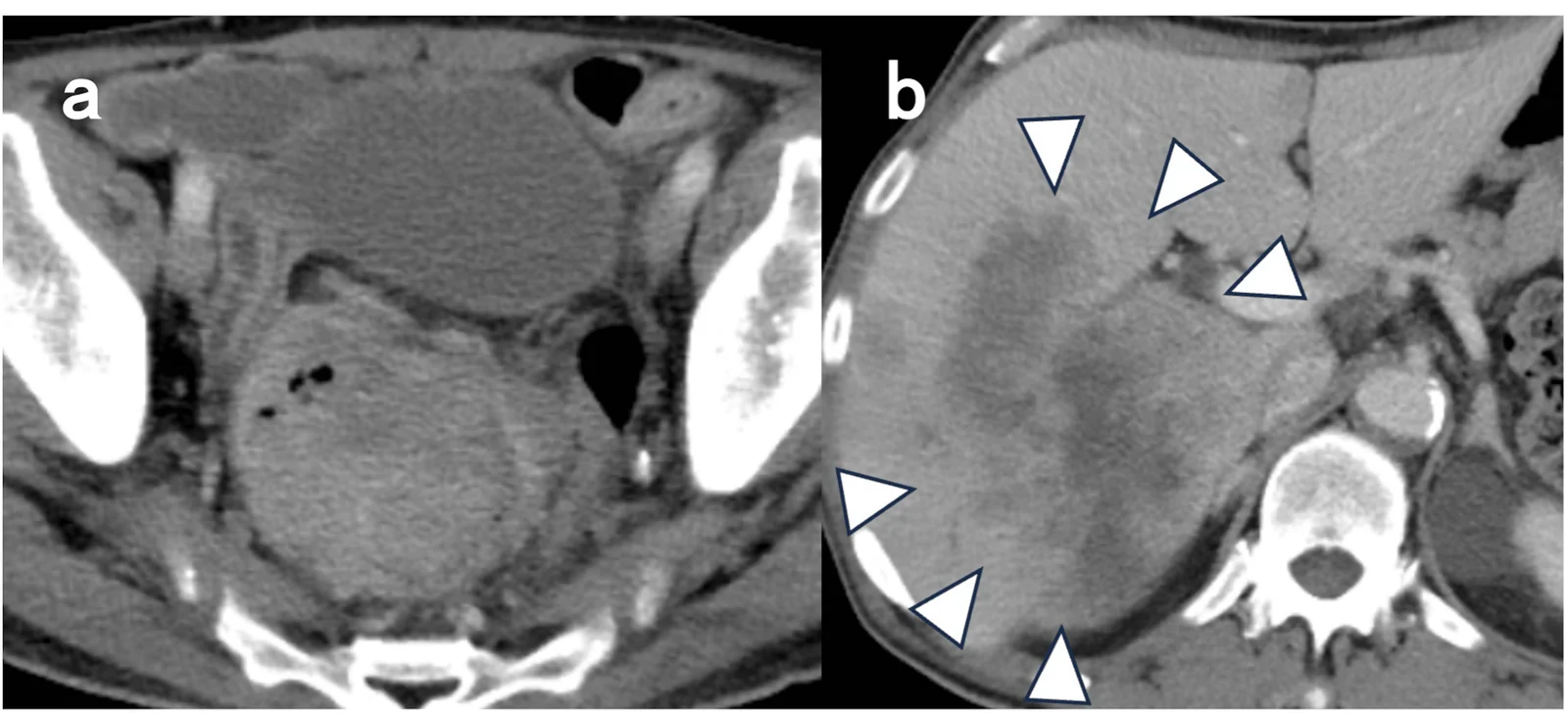
Contrast-enhanced computed tomography images before pembrolizumab treatment (a) Axial contrast-enhanced computed tomography image of the pelvis showing circumferential, contrast-enhanced wall thickening of the sigmoid colon. (b) Axial contrast-enhanced computed tomography image of the upper abdomen showing multiple hypoattenuating lesions in the right hepatic lobe. Arrowheads indicate the liver metastases.

**Figure 4:**
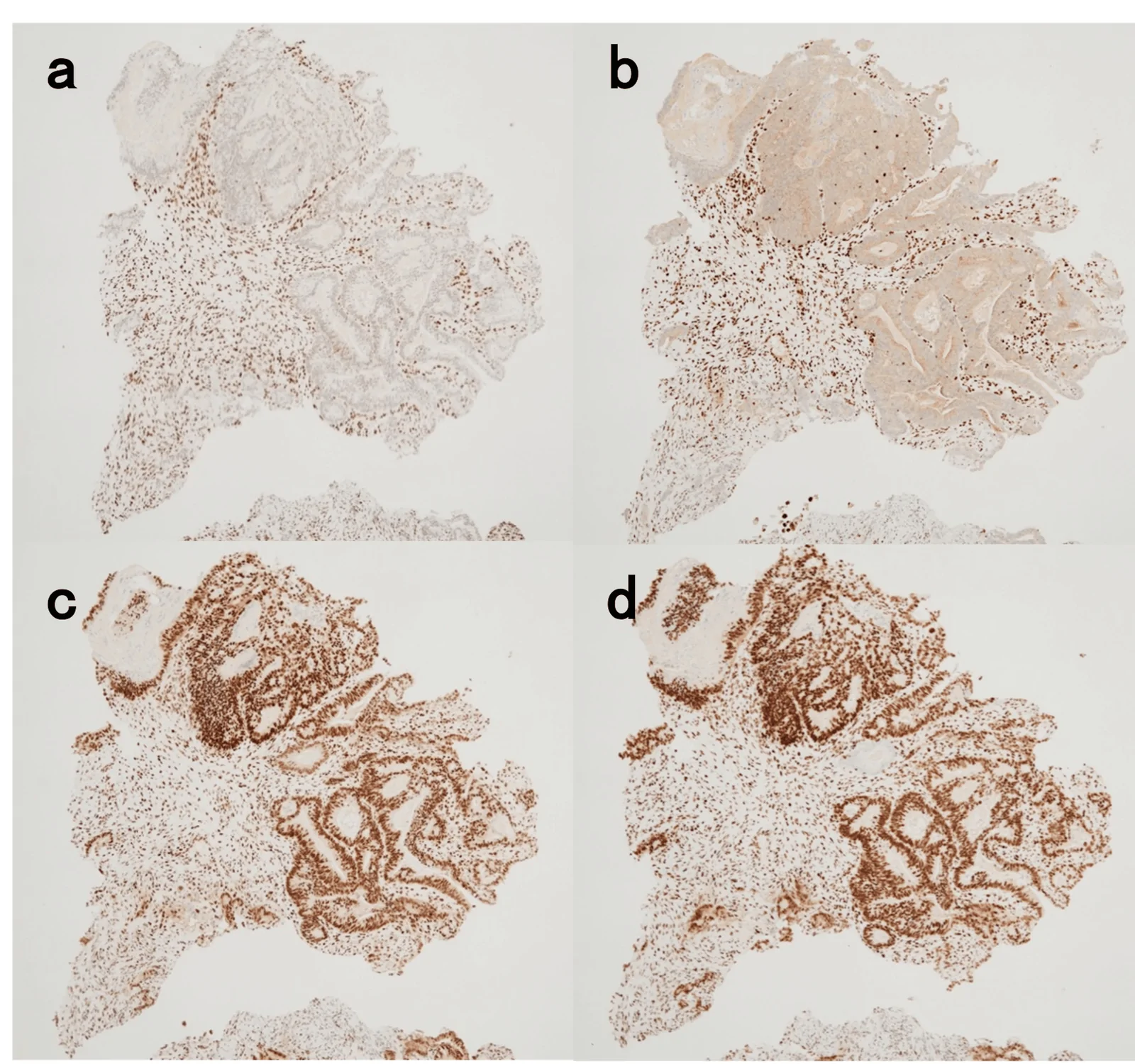
Immunohistochemical staining for mismatch repair proteins (×40) (a) MLH1, (b) PMS2, (c) MSH2, and (d) MSH6. Tumor cells showed loss of MutL homolog 1 (MLH1) and PMS1 homolog 2 (PMS2) expression, with preserved MutS homolog 2 (MSH2) and MutS homolog 6 (MSH6) expression, consistent with deficient mismatch repair status.

A 70-year-old man with a history of diabetes mellitus and hypertension presented with anemia detected during routine blood testing. Lower gastrointestinal endoscopy revealed a circumferential tumor in the sigmoid colon (Figure 1). Biopsy confirmed sigmoid colon adenocarcinoma (Figure 2). Because of bowel stenosis caused by the tumor, the patient was referred for further treatment. Laboratory findings showed anemia with a hemoglobin level of 9.6 g/dL. Tumor markers were markedly elevated at initial presentation: carcinoembryonic antigen (CEA) of 595.4 ng/mL and carbohydrate antigen 19-9 (CA19-9) of 576.8 U/mL. The initial laboratory findings are summarized in Table 1. Contrast-enhanced computed tomography (CT) revealed contrast-enhanced wall thickening in the sigmoid colon (Figure 3a) and multiple hypoattenuating lesions in the right hepatic lobe (Figure 3b). Gadolinium ethoxybenzyl diethylenetriamine pentaacetic acid-enhanced magnetic resonance imaging (Gd-EOB-DTPA-enhanced MRI) confirmed multiple liver metastases. No obvious lymph node metastases were identified. The patient was diagnosed with sigmoid colon cancer with multiple liver metastases, cT4aN0M1a(H3), cStage IVa [9]. Immunohistochemical analysis of mismatch repair proteins revealed loss of MLH1 and PMS2 expression (Figures 4a, 4b), whereas MSH2 and MSH6 expression was preserved, confirming dMMR (Figures 4c, 4d). HER2 was negative, and no mutations were detected in Kirsten rat sarcoma virus (KRAS), neuroblastoma RAS viral oncogene homolog (NRAS), or B-Raf proto-oncogene, serine/threonine kinase (BRAF). Because of bowel obstruction, laparoscopic transverse colostomy was performed. Although simultaneous resection of the primary tumor and liver metastases was initially considered, preoperative assessment revealed triple-vessel coronary artery disease. Therefore, coronary revascularization was prioritized, and immunotherapy was initiated concurrently. 

**Table 1:** Initial laboratory findings at presentation The table summarizes the patient's initial laboratory test results at the time of presentation. Reference ranges are based on the standards of our institution.

Parameters	Patient Values	Reference Range
White blood cell count	8.8	3.3-8.6 × 10^3^/μL
Neutrophils	78.0	40.0-70.0%
Hemoglobin	9.6	13.7-16.8 g/dL
Hematocrit	32	40.7-50.1%
Platelet count	66.2	15.8-34.8 × 10⁴/μL
C-reactive protein	7.27	<0.14 mg/dL
Total protein	6.4	6.6-8.1 g/dL
Albumin	2.5	4.1-5.1 g/dL
Total bilirubin	0.4	0.4-1.5 mg/dL
Aspartate aminotransferase	24	13-30 U/L
Alanine aminotransferase	24	10-42 U/L
Alkaline phosphatase	383	38-113 U/L
Lactate dehydrogenase	275	124-222 U/L
γ-Glutamyl transpeptidase	151	13-64 U/L
Blood urea nitrogen	7	8-20 mg/dL
Creatinine	0.46	0.65-1.07 mg/dL
Estimated glomerular filtration rate	134.6	≥60 mL/min/1.73 m²
Sodium	134	138-145 mmol/L
Potassium	5.0	3.6-4.8 mmol/L
Carcinoembryonic antigen	595.4	≤5.0 ng/mL
Carbohydrate antigen 19-9	576.8	≤37.0 U/mL

Pembrolizumab (200 mg) was administered every three weeks for six cycles. Percutaneous coronary intervention with drug-eluting stent placement was performed for the coexisting ischemic heart disease. Following percutaneous coronary intervention, aspirin 100 mg daily was continued without interruption throughout the perioperative period, including the subsequent surgical procedures.

During pembrolizumab treatment, immune-related endocrine adverse events developed. At the time of the third cycle, approximately six weeks after initiation of pembrolizumab, deterioration in glycemic control raised concern for immune-mediated insulin-dependent diabetes mellitus, and insulin therapy was initiated. Adrenal insufficiency subsequently developed after the fifth cycle, approximately three months after initiation of pembrolizumab, and oral hydrocortisone replacement was started. Both conditions were adequately controlled with insulin and glucocorticoid replacement, respectively. Pembrolizumab was completed after the sixth cycle and was not resumed thereafter.

**Figure 5:**
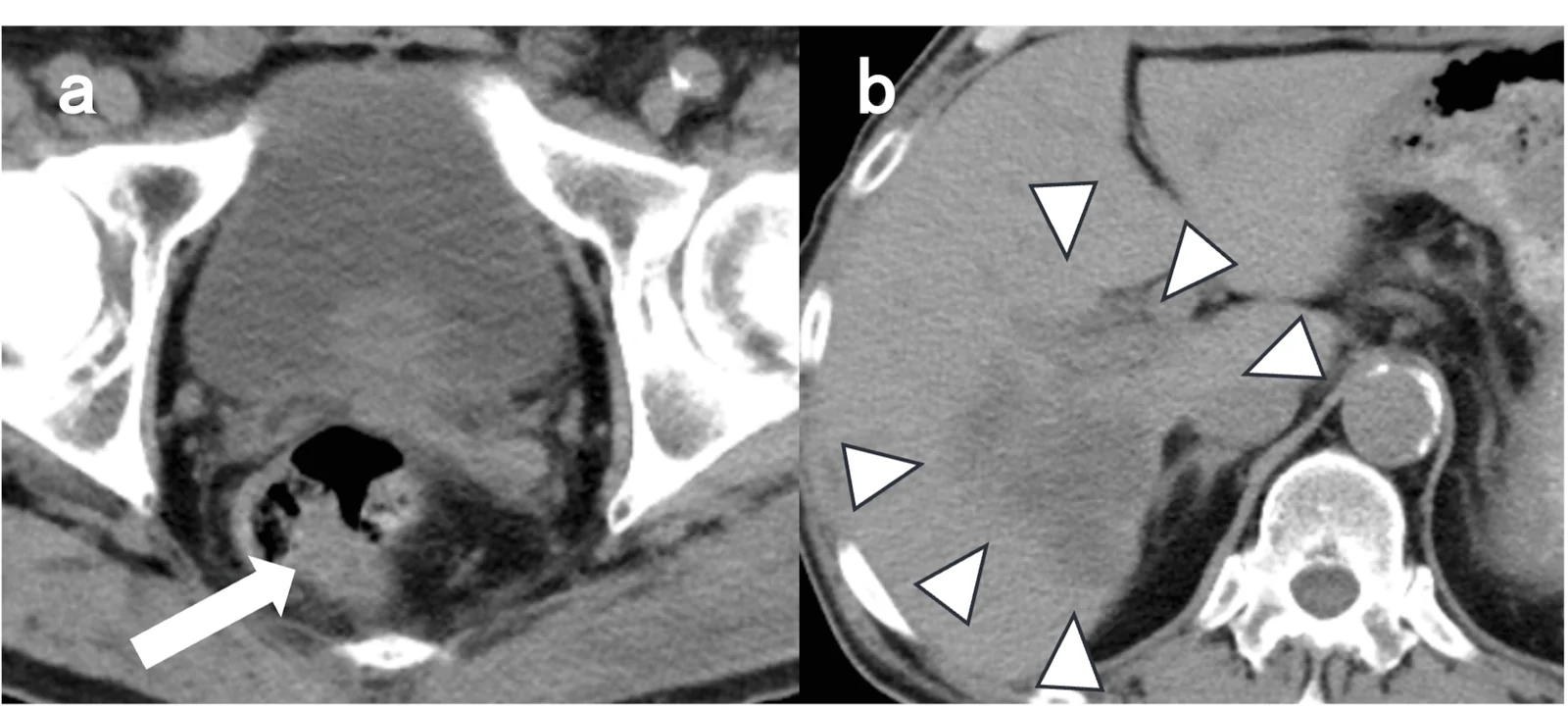
Contrast-enhanced computed tomography images after pembrolizumab treatment (a) Axial contrast-enhanced computed tomography image of the pelvis showing marked regression of the sigmoid lesion, although residual wall thickening with contrast enhancement persisted. The arrow indicates the residual wall thickening of the sigmoid colon. (b) Axial contrast-enhanced computed tomography image of the upper abdomen showing five hypoattenuating lesions in the right hepatic lobe. The dominant lesion, measuring 6.5 cm in maximum diameter, was located in segment 6 near the bifurcation of the anterior and posterior portal venous branches. Additional lesions included a 15-mm lesion near the origin of the right hepatic vein and a 10-mm subcapsular lesion on the surface of segments 5, 6, and 7. All lesions showed uniform hypoattenuation without evident contrast enhancement. Arrowheads indicate the hypoattenuating hepatic lesions.

**Figure 6:**
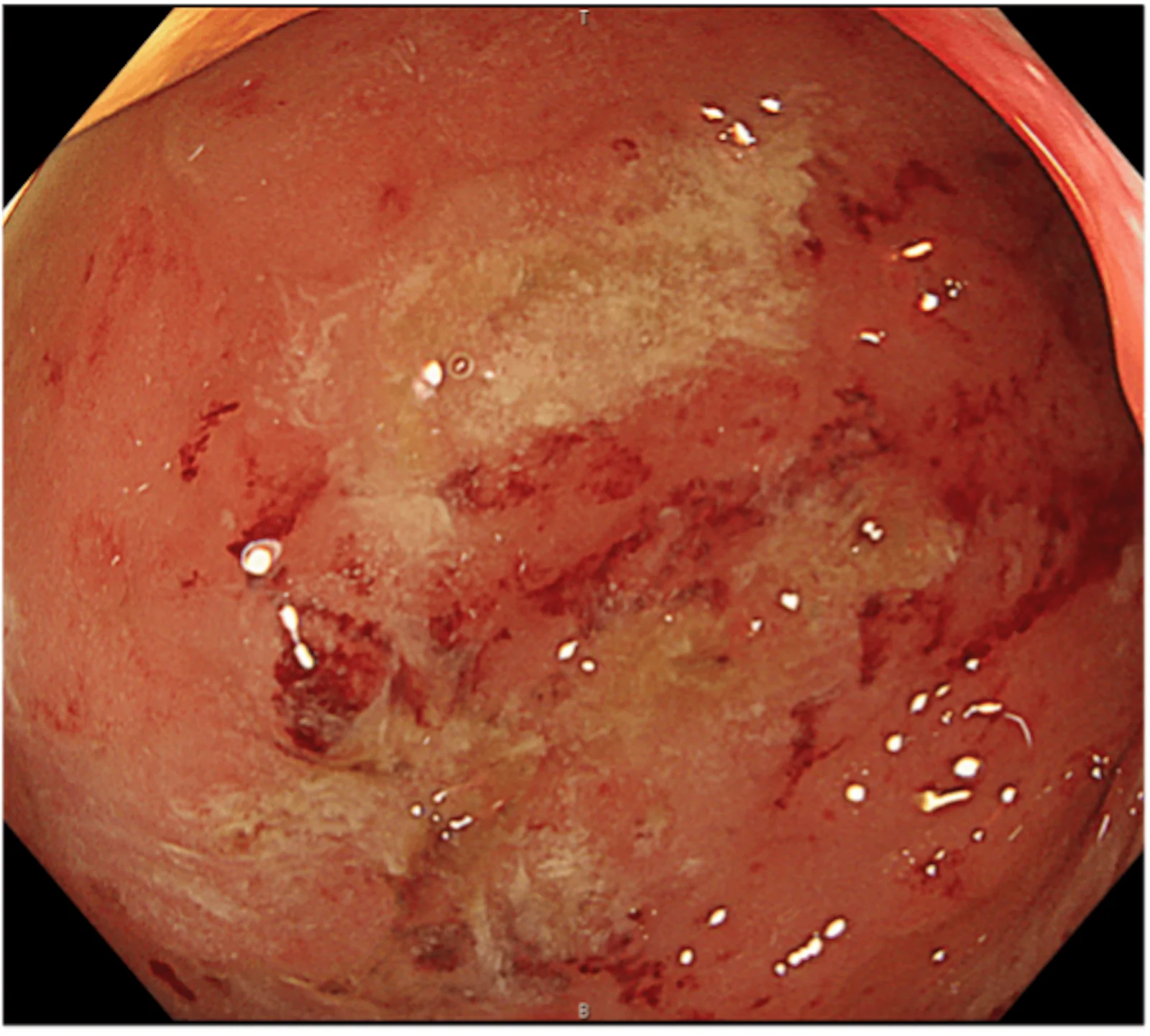
Lower gastrointestinal endoscopic image after pembrolizumab administration At the same location, the tumor was reduced in size and had become scarred.

After pembrolizumab treatment, tumor markers decreased markedly (CEA: 2.9 ng/mL; CA19-9: 4.6 U/mL). The chronological changes in serum tumor markers are summarized in Table 2. Imaging demonstrated marked shrinkage of both the primary and liver lesions. Although near-complete response was observed endoscopically, residual wall thickening of the primary lesion persisted on CT. The largest hepatic lesion decreased from approximately 10.0 cm to 6.5 cm in maximum diameter, corresponding to a 35% reduction, although substantial residual morphological abnormalities remained visible on CT (Figure 5). Colonoscopy revealed tumor shrinkage and scarring in the sigmoid colon (Figure 6). After improvement in cardiac function, robot-assisted sigmoid colectomy was performed four months after treatment initiation. 

**Table 2:** Chronological changes in serum tumor markers during treatment Chronological changes in serum carcinoembryonic antigen (CEA) and carbohydrate antigen 19-9 (CA19-9) levels during treatment. Tumor marker levels decreased markedly following pembrolizumab therapy and remained low before and after surgical resection.

Time point	CEA (ng/mL)	CA19-9 (U/mL)
Initial presentation	595.4	576.8
Before pembrolizumab	1187.5	1541.8
During pembrolizumab treatment	51.4	33.6
Before primary tumor resection	4.2	87.8
Before liver resection	2.9	6.2
After hepatectomy	2.7	4.6

**Figure 7:**
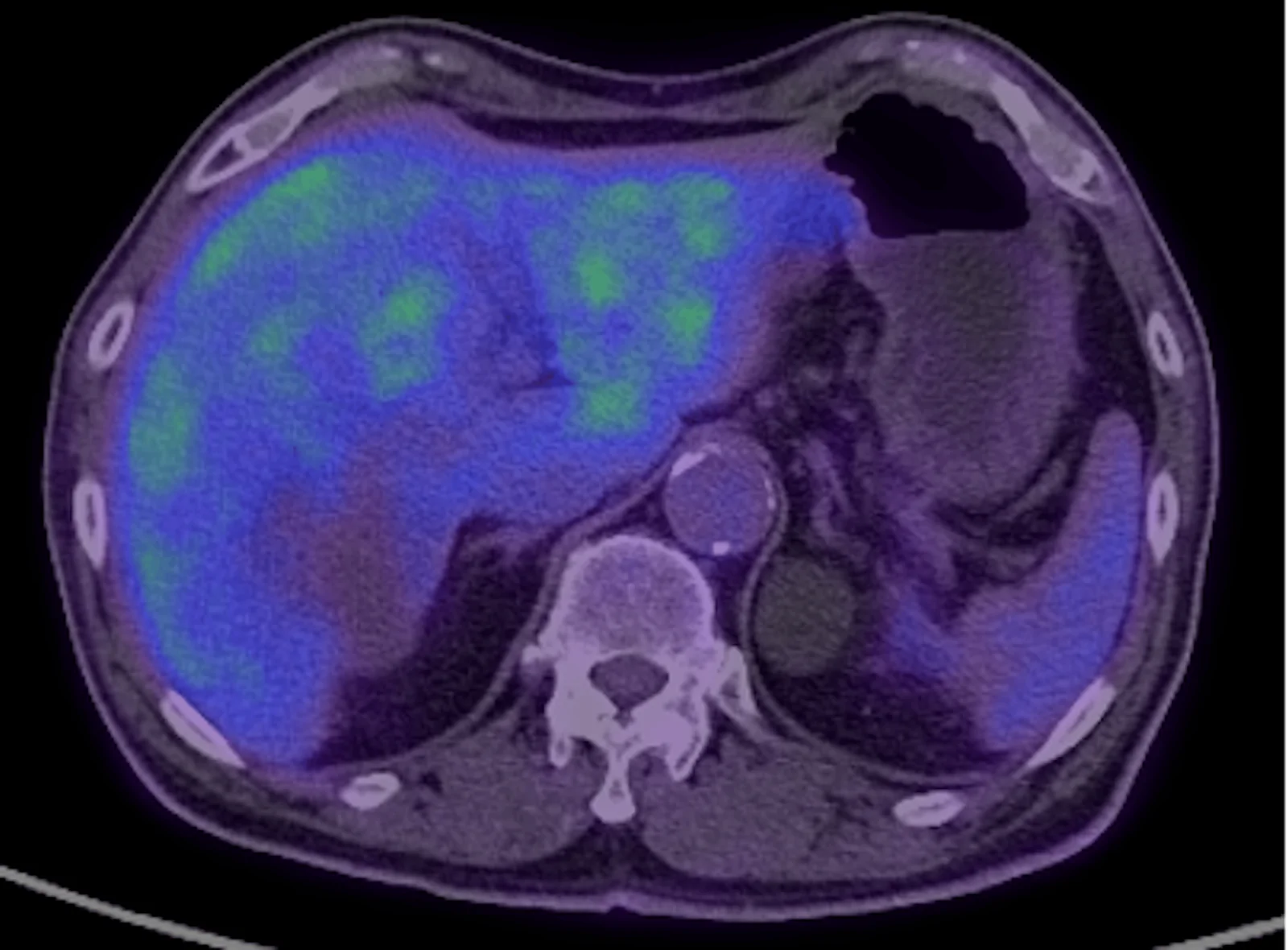
Axial positron emission tomography/computed tomography (PET/CT) image of the upper abdomen after six cycles of pembrolizumab and before hepatic resection PET/CT performed after six courses of pembrolizumab and before hepatic resection demonstrating markedly decreased or absent FDG uptake in the hepatic lesions, suggestive of post-treatment changes and diminished tumor metabolic activity.

**Figure 8:**
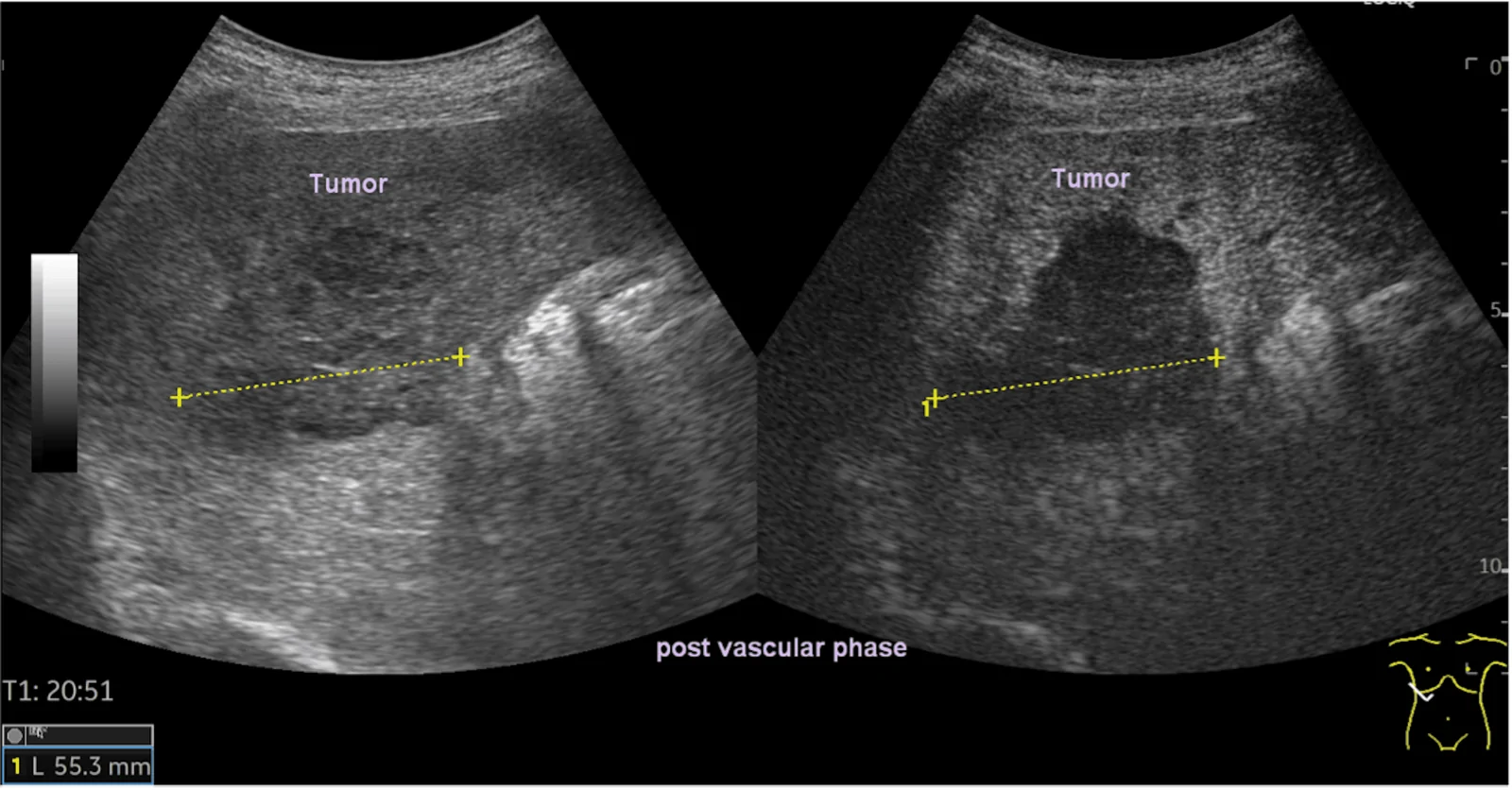
Contrast-enhanced ultrasound (CEUS) CEUS identified four metastatic lesions in the posterior segments of the right hepatic lobe. The hypoechoic tumor in segment 7 showed only faint spotty peripheral vascularity, with no discernible internal vascular structures, suggesting markedly reduced tumor viability following immunotherapy.

Preoperative positron emission tomography/CT (PET/CT) showed no abnormal fluorodeoxyglucose uptake at the primary site and no evidence of local recurrence. The hepatic lesions demonstrated low fluorodeoxyglucose uptake, consistent with post-treatment changes and low metabolic activity (Figure 7). Gd-EOB-DTPA-enhanced MRI revealed multiple hypovascular lesions predominantly in the posterior segments, T2 hyperintensity, and lack of diffusion restriction on diffusion-weighted imaging, with increased apparent diffusion coefficient values, all suggesting reduced tumor viability. Contrast-enhanced ultrasound identified four metastatic lesions in the right hepatic lobe; notably, the segment 7 lesion showed only faint peripheral vascularity without internal flow, suggesting decreased viability (Figure 8).

**Figure 9:**
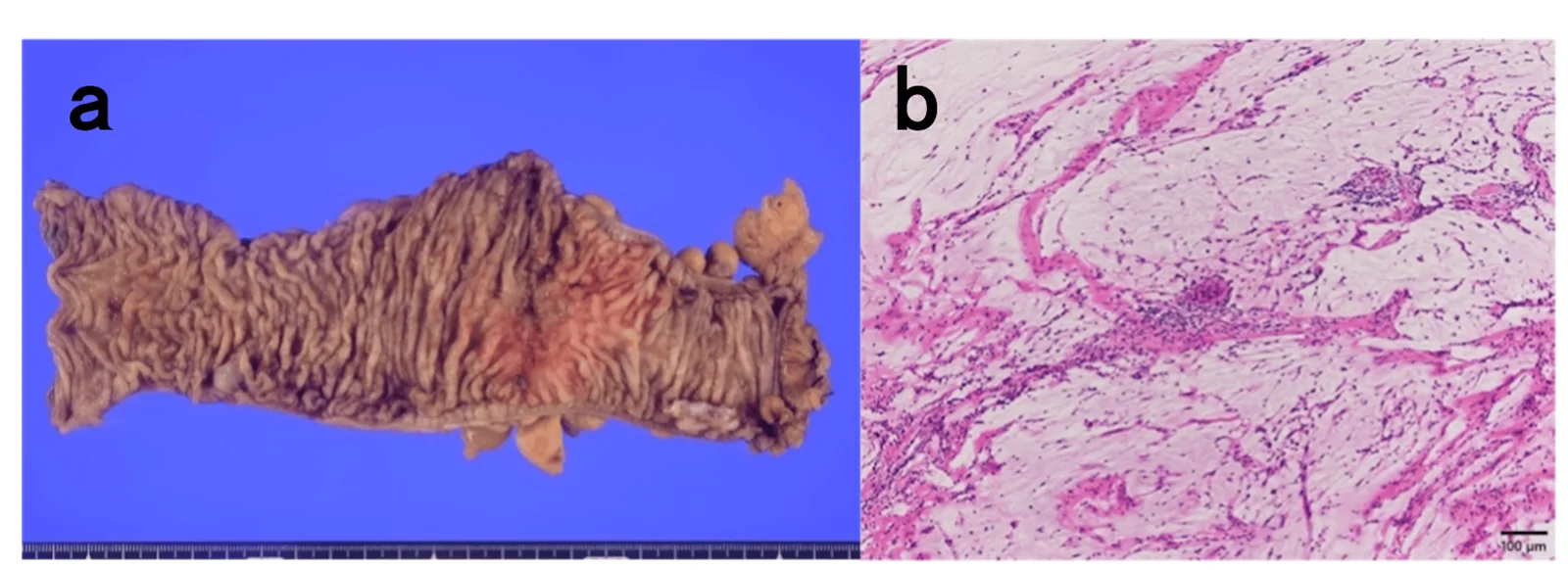
Macroscopic and histopathological findings of the primary tumor (a) Macroscopic appearance of the resected primary lesion. The tumor margin is indistinct, and a mucinous to yellowish-white lesion is observed. (b) Histopathological findings of the primary tumor (hematoxylin and eosin staining, ×10), showing mucin pools extending to the subserosal layer, with cholesterol and hemosiderin deposition, focal calcifications, mild to moderate lymphocytic infiltration, foreign body giant cells, clusters of foamy macrophages, and fibrosis.

**Figure 10:**
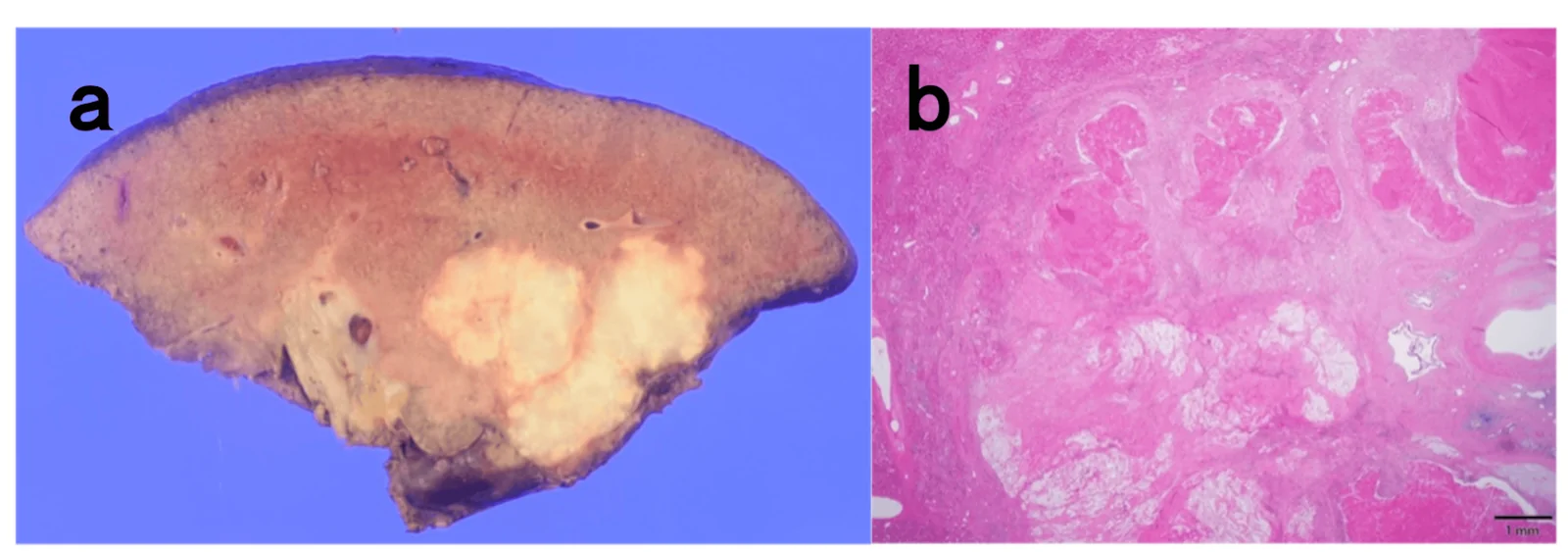
Macroscopic and histopathological findings of the liver metastases (a) Macroscopic appearance of the resected liver metastases. The cut surface shows multiple lobulated tumors ranging in color from whitish to yellowish-white. (b) Histopathological findings of the liver metastases (hematoxylin and eosin staining, ×1.25), showing coagulative necrosis and myxoid changes accompanied by cholesterol clefts and infiltration of lymphocytes and histiocytes. No viable tumor cells are identified in the examined sections.

Seven months after initiation of immunotherapy, laparoscopic right hepatectomy was performed. Histopathological examination of the primary tumor revealed mucin pools extending to the subserosal layer, accompanied by cholesterol and hemosiderin deposits, microcalcifications, lymphocytic infiltration, foamy macrophages, foreign body giant cells, and fibrosis. No viable cancer cells were identified (Figure 9). The pathological findings were ypT0, ypN0 (0/30), with negative proximal and distal resection margins. According to the Japanese Classification of Colorectal, Appendiceal, and Anal Carcinoma, the histological response was classified as Grade 3, corresponding to a pathological complete response [9]. Similarly, the resected liver metastases exhibited features of tumor regression, including coagulative necrosis, myxoid changes, cholesterol clefts, and inflammatory cell infiltration (Figure 10). No viable tumor cells were detected in the examined sections, confirming pCR in the liver metastases as well. The patient remains recurrence-free five months after surgery without adjuvant therapy and is undergoing regular CT surveillance.

## Discussion

This case represents a clinically informative example of pCR in both the primary tumor and liver metastases after preoperative pembrolizumab for stage IVa dMMR sigmoid colon cancer. Of particular importance, residual disease was still strongly suspected on preoperative imaging and macroscopic inspection, especially in the liver, yet histopathological examination revealed no viable tumor cells at either site. Thus, this case does not merely illustrate a near-complete response, but rather a striking discrepancy between persistent morphological abnormalities and true pathological eradication of the tumor.

In recent years, the advent of ICIs has driven a major therapeutic shift in dMMR/MSI-H CRC. In the neoadjuvant setting, mainly for resectable non-metastatic dMMR CRC, previous studies have demonstrated remarkably high pathological response rates, with pCR achieved in a substantial proportion of resected cases [6,7,10]. These outcomes exceed those expected with conventional cytotoxic chemotherapy and support the potential of immunotherapy as a curative-intent strategy in selected patients. However, as the efficacy of ICIs becomes increasingly evident, accurate post-treatment assessment has become a critical issue because conventional morphology-based evaluation does not necessarily reflect the true viability of residual lesions after immunotherapy.

The present case clearly illustrates this challenge. Although the primary lesion regressed endoscopically and the liver metastases showed markedly reduced metabolic activity, CT still demonstrated substantial residual structural abnormalities suggestive of persistent tumor burden. In particular, the hepatic lesions remained clearly recognizable as large residual masses on cross-sectional imaging and were also grossly identifiable at resection, creating a strong clinical impression of persistent metastatic disease. Nevertheless, histopathological examination revealed only treatment-related changes, including necrosis, fibrosis, mucin pools, inflammatory cell infiltration, cholesterol clefts, and foamy macrophages, without any viable tumor cells.

Such discordance between radiological or macroscopic findings and pathological response after ICI therapy has been reported previously [4,6,11]. In particular, a recent meta-analysis by Xie et al. showed that the overall discordance rate between radiological and pathological responses after neoadjuvant immunotherapy in dMMR/MSI-H CRC reached 59.6%, and was higher in colon cancer than in rectal cancer [11]. These findings indicate that, especially in colon cancer, pCR may coexist with morphologically apparent residual lesions, highlighting the limitations of radiological assessment alone. Immune-mediated regression can leave fibrosis, necrosis, mucinous degeneration, and inflammatory infiltrates, all of which may mimic residual tumor on CT, MRI, endoscopy, and even intraoperative inspection. Therefore, response evaluation based solely on RECIST-oriented morphological imaging may be insufficient in the setting of immunotherapy [12]. The present case further supports this concern, because lesions that appeared morphologically residual contained no viable tumor cells on histopathological examination.

For this reason, assessment of treatment response after preoperative ICI should move beyond single-modality judgment. Accumulating evidence suggests that response in dMMR CRC should be interpreted using a multimodal approach that integrates morphology, metabolism, tissue characteristics, and endoscopic findings, rather than relying on size change alone [8,11]. In the present case, CT suggested persistent residual disease, whereas PET/CT showed no meaningful metabolic activity, and MRI and contrast-enhanced ultrasound suggested markedly reduced tumor viability. The final pathological findings indicate that these modalities provided complementary rather than redundant information, collectively reflecting the biological status of the tumor after immunotherapy.

Among these modalities, PET/CT may be particularly valuable. In several malignancies, PET/CT has been reported to contribute to response assessment during ICI therapy by evaluating residual metabolic activity rather than morphology alone [13,14]. However, in dMMR/MSI-H CRC, reports incorporating PET into post-immunotherapy assessment remain limited, and its role has not yet been standardized. In our case, the absence of significant fluorodeoxyglucose uptake in the hepatic lesions was more concordant with the final pathological findings than the persistent structural abnormalities observed on CT or gross examination. This suggests that PET/CT may complement morphology-based imaging in cases where lesions remain visible despite profound biological response.

Another important implication of this case concerns future organ-preserving strategies, including watch-and-wait. In rectal cancer, watch-and-wait after clinical complete response has gained a certain degree of acceptance in carefully selected patients [15,16]. In contrast, watch-and-wait for colon cancer or metastatic lesions after immunotherapy has not been established. The present case raises this possibility because pCR was achieved not only in the primary tumor but also in liver metastases, despite the persistence of morphologically suspicious lesions. At the same time, this case also highlights why immediate expansion of watch-and-wait would be challenging: when CT and gross findings continue to suggest sizable residual disease, clinicians currently have limited confidence in omitting resection.

Further development of watch-and-wait strategies in dMMR CRC will require more reliable criteria to distinguish immune-related regression or residual scar from viable tumor, clarification of the oncological safety of nonoperative management for metastatic lesions, and standardized multimodal response assessment protocols potentially incorporating endoscopy, CT, MRI, PET/CT, and emerging biomarkers. Therefore, although this case suggests the future potential of organ preservation, it should be regarded as hypothesis-generating rather than practice-changing at present.

Given the achievement of pCR in both the primary tumor and liver metastases and the lack of an established benefit of postoperative systemic therapy in this setting, no adjuvant therapy was administered after surgery. In addition, the patient had developed insulin-dependent diabetes and adrenal insufficiency as irAEs. Endocrine irAEs may result in persistent endocrine dysfunction and require long-term or lifelong hormone replacement [17]. In the present case, both conditions were well controlled with self-administered insulin and oral glucocorticoid replacement and were therefore clinically manageable. Nevertheless, the need for continued treatment and monitoring, as well as the potential burden of further therapy, was taken into consideration when determining the postoperative management strategy. Thus, even in patients who achieve a profound antitumor response, the potential long-term burden of endocrine toxicity should be balanced against the therapeutic benefit of immune checkpoint inhibition.

A limitation of this case is the relatively short follow-up period of five months after surgery. Therefore, the long-term durability of the pathological response and oncological outcome remain uncertain, and longer follow-up is required.

Overall, this case highlights two clinically important points. First, pCR can be achieved even in stage IV dMMR CRC with liver metastases after pembrolizumab. Second, and perhaps more importantly, morphologically apparent residual lesions after ICI therapy do not necessarily indicate persistent viable tumor. In particular, lesions appearing as substantial residual masses on CT may represent post-treatment change rather than active cancer. As preoperative immunotherapy becomes more widely applied, accumulation of similar cases and prospective studies will be essential to refine post-treatment assessment, determine surgical indications, and explore whether selected patients may eventually be managed safely with organ-preserving approaches.

## Conclusions

This case demonstrated pathological complete response in both the primary tumor and liver metastases after preoperative pembrolizumab for stage IVa dMMR sigmoid colon cancer. Residual morphological abnormalities after immune checkpoint inhibitor therapy may represent treatment-related changes rather than viable tumor. However, this single case does not establish optimal response assessment methods, surgical indications, or the safety of organ-preserving strategies. At present, when residual lesions remain visible on imaging, pathological confirmation remains the most reliable method for determining true complete response. Therefore, watch-and-wait or other nonoperative strategies should be regarded as hypothesis-generating rather than practice-changing until reliable criteria for identifying pathological complete response without resection are established. Further accumulation of similar cases and longer follow-up are needed to refine response assessment and determine appropriate surgical indications after immunotherapy in dMMR CRC.
